# Supporting women undergoing radiotherapy for cervical cancer: A pilot intervention

**DOI:** 10.4102/hsag.v30i0.2815

**Published:** 2025-04-23

**Authors:** Annah Mosalo, Johanna E. Maree

**Affiliations:** 1 Department of Health Studies, College of Human Sciences, University of South Africa, Pretoria, South Africa; 2 Department of Nursing Education, Faculty of Health Sciences, University of the Witwatersrand, Johannesburg, South Africa

**Keywords:** cervical cancer, support needs, women, radiotherapy, intervention, pilot study

## Abstract

**Background:**

Cervical cancer is the second most common cancer among women in South Africa. Treatment is tailored but external beam radiation and brachytherapy with or without concomitant chemotherapy are commonly used.

**Aim:**

This study aimed to pilot test a support programme for women receiving curative radiotherapy for cervical cancer.

**Setting:**

The Radiation Oncology Department at an academic hospital in the Gauteng province.

**Methods:**

An intervention design and pre-test post-test approach was used. The primary outcome was perceived social support and the secondary outcome was quality of life (QoL). Census sampling entered 56 women in the programme but only 15 completed it. The Berlin Social Support Scale (BSSS) and EORTC QOQ-CX24 served as data collection instruments. Descriptive statistics were used to analyse the data using a completer only approach.

**Results:**

The majority of both the pre-intervention (*n* = 56) and post-intervention (*n* = 15) groups were older than 40 years (62.5%, *n* = 35 and 73.4%, *n* = 11, respectively). Most of the support categories except for ‘support seeking’ showed statistical significant differences before and after the programme. Symptom experience had the highest mean score of the symptoms scales both before and after the programme (*M* = 50.7 and 41.8, respectively).

**Conclusion:**

Positive results were obtained in terms of support, but QoL did not show the same trend. However, it would be feasible to refine the programme and conduct a second pilot test.

**Contribution:**

Our study seems to be the first of its kind and illustrates the positive influence a support programme can have on the lives of women receiving radiotherapy for cervical cancer.

## Introduction

Cervical cancer is the fourth most common cancer in women worldwide, and the fourth most common cause of cancer deaths in women in the world (Sung et al. 2021). According to the Framework for the implementation of the global strategy to accelerate the elimination of cervical cancer as a public health problem in the World Health Organization (WHO) African Region (2021), the disease ranked highest with 110 775 new cancer cases and highest number of deaths (72 705) in 2020. The burden of cervical cancer is not equally distributed as in 2018, 19 of the 20 countries with the highest burden formed part of sub-Saharan Africa (World Health Organization Regional Office for Africa 2021). In South Africa, cervical cancer is the second most common cancer after breast cancer; and in 2019, nearly 7000 women were newly diagnosed with the disease of which the greatest majority, more than 80%, were black (National Institute for Communicable Diseases 2022). However according to Sung et al. (2021), South Africa is one of seven countries in sub-Saharan Africa where a steady increase in the incidence of cervical cancer was observed.

Although cervical cancer is linked to the human papilloma virus (HPV), which is sexually transmitted, it is not sufficient to cause the disease on its own and needs co-factors such as human immunodeficiency virus (HIV) and *Chlamydia trachomatis* infections, smoking, the long term use of oral contraception and multiple childbirths (Sung et al. 2021). Cervical cancer is preventable by means of HPV vaccination, ideally administered before exposure to the virus, as it can take 10 to 20 years for precursor lesions to develop into cervical cancer. Screening and treatment of pre-invasive lesions are also used as preventative measures (World Health Organization 2006, 2022). Women who are HIV positive are at a sixfold higher risk to develop cervical cancer and develop this disease 10 years earlier than those who are HIV negative (Snyman 2013; World Health Organization 2022).

Irrespective of having access to cervical cancer screening, most South African women are not screened and seek healthcare when they experience cervical cancer related symptoms such as vaginal bleeding and discharge and pain, which are signs of advanced cancer (Snyman 2013). Various challenges are linked to this situation (Moodley et al. 2020; Snyman 2013; Van Schalkwyk, Maree & Wright 2008) resulting in presenting with advanced cervical cancer (Stages IB2 to IVB), most commonly Stage IIIB (National Department of Health 2019; Snyman 2013).

The treatment for advanced cervical cancer is tailored according to the stage of disease, the performance status of the patient and medical co-morbidities. However, women with Stages IIB to IVA cervical cancer are treated with curative intent by means of external beam radiation and brachytherapy with or without concomitant chemotherapy. Those diagnosed with Stage IVB cervical cancer receive either curative or palliative treatment (International Atomic Energy Agency 2012; National Department of Health 2019).

Cancer changes the life of the person diagnosed with it permanently. Similar to other people diagnosed with cancer, women diagnosed with cervical cancer are shocked and scared of the cancer, what the future could hold and death (Binka, Doku & Awusabo-Asare 2017; Maree, Langley & Nqubezelo 2015). Their quality of life is also influenced and their global health status is the lowest when receiving treatment (Sabulei & Maree 2019). These women can also lack knowledge of how cervical cancer is treated, what the side effects could be and have unmet information needs. Treatment also creates fear and is considered as a terrible experience of which brachytherapy is the worst (Dzaka & Maree 2016; Long, Friedrich-Nel & Joubert 2016; Maree & Kaila 2014).

Supporting people diagnosed with cancer through the cancer experience is an integral part of nursing (Bafandeh Zendeh, Hemmati Maslakpak & Jasemi 2022). The contact nurses have with cancer patients varies considerably (Tadman, Roberts & Foulkes 2019) as most patients are treated on an out-patient basis. However, women receiving standard curative treatment for cervical cancer receive radiotherapy on a daily basis, 5 days per week, for approximately 6 weeks. Having extended contact with the patients allows nurses ample opportunities to support them with timely information about their cancer, the treatment and side-effects, how to best manage the side-effects or symptoms they are experiencing as well as providing support with the psychosocial, spiritual and practical challenges (Evans Webb et al. 2021). Patient needs differ and whether the identified needs can be considered to be the support needs of South African women is not clear, as these women face unique challenges such as poverty, a lack of awareness of cervical cancer, traditional beliefs, medicine and healers, and challenges related to the healthcare system (Maree, Holtslander & Maree 2021; Moodley et al. 2020; Snyman 2013; Van Schalkwyk et al. 2008). Therefore, our study focussed on the support needs of women treated for cervical cancer in a public hospital in South Africa and answered the following research question: *Would a support programme developed according to the needs of women receiving curative treatment for cervical cancer at an academic hospital in the Gauteng province improve their perception of being supported and their quality of life (QoL)?*

As evident from the research question, this was a multiphased study and this article will report the results of the pilot test to describe whether the support programme developed according to the needs of these women improved their perception of being supported and their QoL. Exploring their support needs and developing the support programme are reported elsewhere.

## Research methods and design

### Design and setting

The authors used an intervention design and pretest-post-test approach to assess the outcomes of the support programme. Intervention studies allow researchers to determine what strategies work best to improve health outcomes (Melnyk & Morrison-Beedy 2012) by pilot testing a newly developed intervention (Conn et al. 2010). Using a pretest-post-tests approach enabled us to measure the variables, in this case perception of being supported and QoL, before and after implementing the support programme in the absence of a control arm (Aggarwal & Ranganathan 2019).

The study setting was an academic hospital in the Gauteng province in South Africa, which provides highly specialised healthcare services to all categories of patients, including those diagnosed with cancer. Surgery, systemic anticancer therapies and radiotherapy are offered as primary treatments. The majority of patients with cancer are treated on an outpatient basis and the radiation oncology department treats approximately 70 patients with cervical cancer per day. In addition to a waiting area, the department has a special room where chemotherapy is administered to patients receiving concomitant chemotherapy as well as a resting room accommodating patients in need of bedrest (Sabulei & Maree 2019). As radiotherapy is administered on a daily basis for 6 consecutive weeks to these patients, the same number of patients are treated per week although the number can vary.

### Procedure

#### Developing and implementing the support programme

Based on the first phase of the greater study investigating the support needs of women treated for cervical cancer and how they would prefer to be supported, the first author developed a support programme. This programme was validated by means of a group of experts in the field of cancer treatment and care, and patients treated for cervical cancer. The programme focussed primarily on informational support, but included emotional support and counselling as the need arose. The programme was implemented in the radiation oncology department of the study setting during April, May and June 2020. Each session was facilitated twice to optimise attendance. The first author facilitated the programme and summarised the information of the previous session before continuing with the scheduled session. Time for questions, comments and sharing of experiences were also allowed. The sessions included the following:

- *Session 1* provided an orientation to the programme including its aims, the different healthcare professionals involved in the treatment and care, and basic information about cancer in general. The programme was named and experiences were shared.
- *Session 2* covered the anatomy of the female reproductive system and organs in relation to the cervix.
- *Session 3* presented information pertaining to screening for cervical cancer. Pap smear kits, cyto brushes and disposable speculums were available for participants to observe and handle, to familiarize themselves to re-assure them that the procedure was not invasive and instruments used will not cause any harm.
- *Session 4* introduced the simulation procedure and treatment field markings. Practical tips on self-care were given and other support services such as hospital transport facilities and accessing temporary social grants were introduced.
- *Session 5* presented detailed information about simulation and the different stages of cervical cancer. The different treatment methods were also discussed.
- *Session 6* discussed the side-effects that the patients could experience in terms of how to identify and manage it. The importance of instrumental support was also discussed.

#### Recruiting the participants

The first author liaised with the Nursing Unit Manager to identify women receiving curative treatment for cervical cancer and invited them to participate in the study. Census sampling (Australian Bureau of Statistics 2023) were used and all the women meeting the inclusion criteria of having a confirmed diagnosis of cervical cancer and treated with radiotherapy were approached to participate in the study. A total of 56 women were enrolled in the programme. This sample size was considered sufficient to give information as to whether the intervention would work (Thabane et al. 2010).

### Data collection and instruments

A researcher developed a demographic data collection sheet and two validated questionnaires, the Berlin Social Support Scale (BSSS) and European Organization for Research and Treatment of Cancer (EORTC) QOQ-CX24 were used with permission. The demographic data sheet asked questions about age group, marital status, cultural group and educational level. The BSSS measures cognitive and behavioural aspects of social support by means of six subscales; perceived support, actually provided and received social support, need for support, support seeking, protective buffering and internal consistency for provided social support. One item presented in combination with ‘actually provided and received social support’, measures satisfaction with the support received. A Likert-type scale is used for the answers where 1 = strongly disagree, 2 = somewhat disagree, 3 = somewhat agree 4 = strongly agree. The scales can be scored by either adding them up or calculating the mean. The internal consistency for the subscales in the validation sample was reported as perceived support, Cronbach’s alpha = 0.83, received social support = 0.83, need for support = 0.63, support seeking = 0.81, protective buffering = 0.82; internal consistency for provided social support in partner sample = 0.75 (Schwarzer & Schulz 2003).

The EORTC QLQ-CX24 measures cervical cancer-specific quality of life by means of 24 questions. This scale consists of three multi-item scales measuring symptom experiences (11 items), body image (3 items) and sexual and/or vaginal functioning (4 items). Six single item scales measures lymphoedema, peripheral neuropathy, menopausal symptoms, sexual worry, sexual activity and sexual enjoyment. The items are graded using a Likert-type of scale with: 1 = not at all, 2 = a little, 3 = quite a bit and 4 = very much. The answers were converted into a 0 to 100 scale as per EORTC QLQ scoring manual (Fayers et al. 2001). The higher the score of the functional scales, the better the function; the lower the score in the symptom scales, the more severe the symptom experience. Multitrait scaling analysis revealed high internal consistency with a Cronbach’s alpha coefficients ranging from 0.72 to 0.87; symptom experience, 0.72; body image, 0.86; sexual and/or vaginal functioning, 0.87 (Greimel et al. 2006).

To collect the pretest data, the first author who is conversant with the languages spoken in the facility administered all three questionnaires at base line and before the participants entered the support programme with the assistance of a field worker. The post-test questionnaires (BSSS and EORTC QOQ-CX24) were administered on the day the participants completed the programme. Data were collected from 56 participants before the programme and from 15 completing the programme resulting in an attrition rate of 73.2%. Reasons for attrition were related to interruption and rescheduling of treatment caused by the breakdown of the radiation machines, failure to adhere to the treatment plan, loss of interest and physical and emotional distress.

### Data management and analyses

The completed questionnaires were placed in an envelope where after it was numbered, and the data were entered into an Excel spread sheet. The data were cleaned, imported into the Statistical Package for the Social Sciences (SPSS) 22 computer program and analysed by means of descriptive statistics. Wilcoxon ranked sum tests were used to calculate significant differences between the variables (Marusteri & Bacarea 2010). As the authors wished to assess the outcomes of the support program when completed in total, the completer only approach (Andrade 2022) was used to analyse the data.

### Ethical considerations

Data collection commenced after ethical approval was obtained from the University of the Witwatersrand by the Human Research Ethics Committee (Medical) of the University (clearance no.:M150272). Permission was also obtained from the hospital management and the Head of the Radiation Oncology Department. Informed consent in writing was obtained from prospective participants after they agreed to participate in the study. A distress protocol applied and counselling, free of costs to the participants, were available to those who became emotionally distressed; however, this service was not used.

## Results

The majority of both the pre-intervention (*n* = 56) and post-intervention (*n* = 15) groups, were older than 40 years (62.5%; *n* = 35 and 73.4%; *n* = 11, respectively); employed (53.6%; *n* = 30 and 73.3%; *n* = 11, respectively) and attended secondary school (51.8%; *n* = 29 and 60%; *n* = 10, respectively). Women in the post intervention group tended to be between the ages 41 and 50 (46%; *n* = 7), single (60%; *n* = 9) with Grade 8 to 12 education (67%, *n* = 10) and employed (73.3%; *n* = 11). The Wilcoxon Rank test indicated a significant large difference between the group who remained in the study and those lost to attrition, *p* ˂ 0.001 (Table 1).

**TABLE 1:** Demographics characteristics of the pre-intervention (*n* = 56) and post- intervention groups (*n* = 15).

Variables	Pre-intervention (*n* = 56)		Post-intervention (*n* = 15)	
	*n*	%	*n*	%
**Age (years)**				
18–25	1	1.8	0	0
26–30	4	7.1	0	0
31–40	11	19.6	3	20.0
41–50	14	25.0	7	46.7
51 and above	21	37.5	4	26.7
Missing	5	8.9	1	6.7
**Marital status**				
Married civil	9	16.1	1	6.7
Married culturally	8	14.3	0	0
Co-habiting	4	7.1	2	13.3
Single	27	48.2	9	60.0
Widowed	6	10.7	1	6.7
Divorced	2	3.6	2	13.3
**Cultural group**				
Ndebele	1	1.8	0	0
North Sotho	3	5.3	1	6.7
South Sotho	10	17.9	3	20.0
Tsonga	2	3.6	0	0
Xhosa	8	14.3	4	26.7
Zulu	19	33.9	4	26.7
Other	13	23.2	3	20.0
**Educational level**				
No formal education	2	3.6	0	0
Grade 1–7	22	39.3	4	26.7
Grade 8–12	29	51.8	10	66.0
Tertiary	3	5.4	1	6.7
**Employment status**				
Employed	30	53.6	11	73.3
Unemployed	14	25.0	4	26.7
Pensioner	11	19.6	0	0
Disability grant	1	1.8	0	0

When investigating social support, perceived emotional support and support seeking had the highest mean score pre-intervention (*M* = 3.6, respectively) while the need for support had the lowest mean score (*M* = 3.2). After the intervention, perceived instrumental support had the highest mean score (*M* = 4.0) while protective buffering had the lowest mean score (*M* = 3.1). The Wilcoxon Rank test indicated a significant difference between the pre-test and post-test variables of five of the six variable groups except for support seeking (*p* = 0.1). When looking at the subscales, the variable ‘I get along best without outside help’ had the lowest mean score before the intervention (*M* = 1.9) while ‘This person criticized me’ had the lowest mean score after the intervention (*M* = 2.3). The details are presented in Table 2.

**TABLE 2:** Social support before and after the support programme (*N* = 15).

Variables	Pre-intervention		Post-intervention		*p*-value
	Total score	Mean ± s.d.	Total score	Mean ± s.d.	
**Perceived emotional support**	217	3.6 ± 0.7	228	3.8 ± 0.4	0.0051*
• There are some people who truly like me	53	3.5 ± 0.9	57	3.8 ± 0.4	
• Whenever I am not feeling well, other people show me that they are fond of me	54	3.6 ± 0.7	57	3.8 ± 0.4	
• Whenever I am sad, there are people who cheer me up	54	3.6 ± 0.7	57	3.8 ± 0.4	
• There is always someone there for me when I need comforting	56	3.7 ± 0.6	57	3.8 ± 0.4	
**Perceived instrumental support**	210	3.5 ± 0.8	237	4.0 ± 0.2	0.0001*
• I know some people upon whom I can always rely on	55	3.7 ± 0.7	60	4.0 ± 0.0	
• When I am worried there is someone who helps me	54	3.6 ± 0.6	60	4.0 ± 0.0	
• There are people who offer me help when I need it	50	3.3 ± 0.7	60	4.0 ± 0.0	
• When everything becomes too much for me to handle, others are there to help me	51	3.4 ± 1.0	57	3.8 ± 0.2	
**Need for support**	168	3.2 ± 1.1	202	3.4 ± 1.0	0.0009*
• When I am down, I need someone who boosts my spirit	53	3.5 ± 0.7	56	3.7 ± 0.4	
• It is important for me always to have someone who listens to me	54	3.6 ± 0.7	55	3.7 ± 0.5	
• Before making any important decisions, I absolutely need a second opinion	50	3.3 ± 1.1	52	3.5 ± 1.0	
• I get along best without any outside help	29	1.9 ± 1.0	39	2.6 ± 1.2	
**Support seeking**	267	3.6 ± 0.8	267	3.6 ± 0.8	0.1000
• In critical situations, I prefer to ask others for their advice	56	3.7 ± 0.8	53	3.5 ± 0.9	
• Whenever I am down, I look for someone to cheer me up again	56	3.7 ± 0.7	53	3.5 ± 0.9	
• When I am worried, I reach out to someone to talk to	51	3.4 ± 1.1	53	3.5 ± 0.8	
• If I don’t know how to handle a situation, I ask others what they would do	52	3.5 ± 0.6	54	3.6 ± 0.7	
• Whenever I need help, I ask for it	52	3.5 ± 0.6	54	3.6 ± 0.7	
**Actually received support**	725	3.5 ± 0.9	756	3.6 ± 0.9	0.0221*
• The person showed me that he or she loves and accepts me	55	3.7 ± 0.6	60	4.0 ± 0.0	
• This person comforted me when I was feeling bad	54	3.6 ± 0.7	58	3.9 ± 0.3	
• This person left me alone	50	3.3 ± 1.0	40	2.7 ± 1.5	
• This person did not show much empathy for my situation	47	3.1 ± 1.2	37	2.5 ± 1.5	
• This person criticised me	46	3.1 ± 1.6	35	2.3 ± 1.4	
• This person made me feel valued and important	54	3.6 ± 0.7	60	4.0 ± 0.0	
• This person expressed concern about my condition	54	3.6 ± 0.7	58	3.9 ± 0.4	
• This person assured me that I can rely completely on him or her	52	3.5 ± 0.7	59	3.9 ± 0.3	
• This person encouraged me not to give up	54	3.6 ± 0.7	60	4.0 ± 0.0	
• This person was there when I needed him or her	52	3.5 ± 1.1	60	4.0 ± 0.0	
• This person took care of many things for me	50	3.3 ± 0.8	58	3.9 ± 0.5	
• This person took care of things I could not manage on my own	51	3.4 ± 0.6	58	3.9 ± 0.5	
• This person helped find something positive in my situation	54	3.6 ± 0.7	58	3.9 ± 0.5	
• This person suggest activities that might distract me	52	3.5 ± 0.7	55	3.4 ± 0.7	
**Protective buffering**	299	3.4 ± 0.9	276	3.1 ± 1.1	0.0002*
• I kept all bad news from him or her	51	3.4 ± 1.1	51	3.4 ± 1.1	
• I avoided everything that could upset him or her	57	3.8 ± 0.6	48	3.2 ± 1.1	
• I showed strength in his or her presence	52	3.5 ± 0.7	49	3.3 ± 1.1	
• I did not let him or her noticed how bad and depressed I really felt	42	2.8 ± 1.1	40	2.7 ± 1.3	
• I avoided any criticism	42	2.8 ± 1.1	40	2.7 ± 1.3	
• I pretended to be very strong although I did not feel that way	55	3.7 ± 0.6	48	3.2 ± 1.1	
**Satisfaction with support received**	53	3.5 ± 0.7	59	3.9 ± 0.3	-
• In general, I am very satisfied with the way this person behaved	53	3.5 ± 0.7	59	3.9 ± 0.3	

*Source:* Schwarzer, R. & Schulz, U., 2003, ‘Soziale Unterstützung Bei Der Krankheitsbewältigung: Die Berliner Social Support Skalen (BSSS)’, *Diagnostica* 49, 73–82.

s.d., standard deviation.

* , significance *p* < 0.05.

When investigating QoL, body image had the highest mean score both before and after the support programme (*M* = 71.9 and *M* = 68.0, respectively). However, both functional subscales (body image and sexual activity) scored less in the post-test. Symptom experience had the highest mean score of the symptoms scales both before and after the programme (*M* = 50.7 and *M* = 41.8, respectively) while peripheral neuropathy had the lowest pretest mean score (*M* = 6.7) and lymphoedema the lowest post-test mean score (*M* = 15.6). The Wilcoxon Rank test did not find any significant difference between the pre-test and post-test variables for body image and symptom experience. The details are presented in Table 3.

**TABLE 3:** Quality of life before and after the support programme (*N* = 15).

Domain	Pre-test	Post-test	*p*-value
	Mean ± s.d.	Mean ± s.d.	
**Functional scale**			
Body image	71.9 ± 8.3	68.0 ± 7.1	0.38*
Sexual activity	26.7 ± 1.8	15.6 ± 0.7	-
Sexual enjoyment	N/A	-	-
Vaginal functioning	N/A	-	-
**Symptom scales**			
Symptom experience	50.7 ± 48.1	41.8 ± 30.9	0.47*
Lymphoedema	46.7 ± 45.6	15.6 ± 43.2	-
Peripheral neuropathy	6.7 ± 6.7	26.7 ± 42.5	-
Menopausal symptoms	40.0 ± 48.4	26.7 ± 26.5	-
Sexual worry	40.0 ± 47.6	34.7 ± 50.0	-

*Source:* Greimel, E.R., Kuljanic Vlasic, K., Waldenstrom, A.C., Duric, V.M., Jensen, P., Singer, S. et al., 2006, ‘The European Organization For Research And Treatment Of Cancer (EORTC) quality-of-life questionnaire cervical cancer module: EORTC QLQ-CX24’, *Cancer* 107, 1812–1822. https://doi.org/10.1002/cncr.22217

s.d., standard deviation; N/A, not applicable.

* , significance *p* < 0.05.

## Discussion

Our sample, being primarily over the age of 40, with the highest percentage 50 years and older, is similar to what Dhokotera et al. (2022) reported when summarising the factors associated with cervical cancer in South Africa. As seen from our study, the highest percentage of the sample was single that was also found in other studies (Kaila & Maree 2018; Sabulei & Maree 2019) and similar to the South African population (STASISTA 2023). Having a spouse or life partner can be beneficial in terms of support but is not guaranteed as Maree, Mosalo and Wright (2013), in a South African study investigating life partner support, found the support women received from their life partner when treated for cervical cancer varied; some were fully supported while others received limited support or were even abandoned. Ndikom, Aluko and Adeoye (2019:89) found a similar trend in terms of the QoL of women treated for breast and gynaecological cancer in Nigeria as the support they received from their spouses did not significantly improve their QoL (Jansen van Rensburg, Maree & Casteleijn 2017).

Despite the availability of the support programme, which was based on the needs of a similar group of women treated in the same facility, the large attrition rate was disappointing. The rescheduling of treatment because of the breakdown of the radiotherapy machines can be considered as exceptional circumstances, but some of the other reasons have also been described in the literature. For instance, Najjemba et al. (2023) in a Ugandan study, found only 12% of the 196 women included in their study, adhered to their scheduled cervical cancer treatment regimens. Stigma in healthcare facilities has also been identified as barrier to seeking care that can improve QoL (Nyblade et al. 2019) and could have also led to attrition. This, however, is mere speculation and should be investigated before conclusions can be made.

In terms of support, most of the categories except for ‘Support seeking’ showed statistical significant differences before and after the programme. It was interesting to find that ‘I get along best without any outside help’, indicating a need for support, scored the lowest in the pretest (*M* = 1.9) and remained one of the lowest scores in the post-test (*M* = 2.6). The exact help needed is not known, however, the study is in contrast to the findings of Uysal et al. (2019), in a study conducted in Turkey, where patients receiving radiotherapy needed support in all aspects of life as the highest percentages of their 260 respondents indicated they experienced fatigue (66%; *n* = 133) and pain (43.2%; *n* = 86).

It was positive to find that all the participants gave the highest score for the categories ‘this person showed she loves and accepts me’ and ‘made me feel valued and important’. Unfortunately this might not be the case when these women return to their communities. When synthesising the experiences of women living with cervical cancer in Africa, found their support systems often failed and rejected them that added to their suffering (Maree et al. 2021). These support systems included their life partners and other family members, community members and even the church. In addition, women are still blamed for their cervical cancer diagnosis and seen as promiscuous (Morse et al. 2023; Shepherd & Gerend 2014), despite evidence that not all women can protect themselves from cervical cancer as their economic situation, fear, physical abuse and helplessness prevent them from negotiating condom use (Maharajh & Haffejee 2021; Maree 2010).

Although no statistically significant differences were found in terms of QoL, the study showed that both functioning scales, body image and sexual activity decreased during the time of treatment. A similar downwards trend was found in terms of body image in the studies conducted in India by Singh et al. (2019) and Dahiya et al. (2016) who measured QoL before and 6 months after treatment. Symptom experience also became worse as treatment progressed. Unfortunately, this trend continued when measured at 6 months after treatment (Dahiya et al. 2016; Singh et al. 2019). Peripheral neuropathy was the exception of the symptoms scales, as this symptom experience became less after the support programme. This trend concurs with the study of Dahiya et al. (2016), but is in contrast with the trend found by Singh et al. (2019).

### Strengths and limitations

This study seems to be first of its kind as the authors could not find other studies evaluating support programmes for cancer patients while receiving radiotherapy. When considering the outcomes of the study, it is important to remember that the study was subjected to various limitations that could influence the results. This includes history, maturation and testing (Knapp 2016; Kumanyika, Parker & Sim 2010). Small studies can also produce false positive results (Hackshaw 2008). Therefore, the results should be considered with great caution. However, this was a pilot intervention study testing if a newly developed programme would work.

## Conclusion

This pilot study pilot tested a support programme for women receiving curative radiotherapy for cervical cancer at an academic hospital in Gauteng. The primary outcome was perceived social support and secondary outcome was QoL. Both outcomes were achieved. Positive results were obtained in terms of the perception of receiving social support, but the functional and symptom scales of QoL did not show the same trend. Therefore, it would be feasible to refine the programme and pilot test the refined programme. For a more accurate measurement of QoL, the EORTC QLQ-C30, a questionnaire developed to assess the QoL of cancer patients could be used in combination with the EORTC QLQ-CX24.
